# *In vitro* determination of the anti-aging potential of four southern African medicinal plants

**DOI:** 10.1186/1472-6882-13-304

**Published:** 2013-11-05

**Authors:** Gugulethu Ndlovu, Gerda Fouche, Malefa Tselanyane, Werner Cordier, Vanessa Steenkamp

**Affiliations:** 1 Natural Product Chemistry Group, Biosciences Unit, Council for Scientific and Industrial Research, P.O. Box 395, Pretoria (0001), South Africa; 2 Department of Chemistry, University of Free-State, P.O. Box 339, Bloemfontein (9300), South Africa; 3 Molecular and Biomedical Technologies, Biosciences Unit, Council for Scientific and Industrial Research, P.O. Box 395, Pretoria (0001), South Africa; 4 Department of Pharmacology, University of Pretoria, Private Bag X323, Arcadia (0007), South Africa

**Keywords:** Anti-aging, Anti-collagenase, Anti-elastase, Anti-hyaluronidase, Anti-oxidant, Medicinal plants

## Abstract

**Background:**

Aging is an inevitable process for all living organisms. During this process reactive oxygen species generation is increased which leads to the activation of hyaluronidase, collagenase and elastase, which can further contribute to skin aging. Four southern African medicinal plants; *Clerodendrum glabrum*, *Schotia brachypetala*, *Psychotria capensis* and *Peltophorum africanum,* were investigated to assess their anti-aging properties.

**Methods:**

Anti-elastase, anti-collagenase and anti-hyaluronidase activities of twenty-eight samples, consisting of methanol and ethyl acetate extracts of the four plants, were determined using spectrophotometric methods. Radical scavenging activity was determined by the ability of the plant extracts to scavenge the ABTS^•+^ radical.

**Results:**

The majority of the samples in the anti-elastase assay and nine in the anti-collagenase assay showed more than 80% inhibition. The ethyl acetate extract of *S. brachypetala* bark and leaves of *P. capensis* inhibited elastase activity by more than 90%. The methanol extract of *S. brachypetala* bark contained the highest anti-hyaluronidase activity (75.13 ± 7.49%) whilst the ethyl acetate extract of *P. africanum* bark exhibited the highest antioxidant activity (IC_50_: 1.99 ± 0.23 μg/ml).

**Conclusion:**

The free radical scavenging activity and enzyme inhibitory activity of the plant extracts investigated suggests that they can help restore skin elasticity and thereby slow the wrinkling process. *P. africanum* was the plant with the most promising activity and will be subjected to further testing and isolation of the active compound/s.

## Background

Aging is an inevitable process for all living organisms. In humans the skin is the tissue most markedly affected. Two types of skin aging exist: age-dependent/chronological aging and premature aging/photoaging [1]. The latter is caused by extrinsic factors and includes signs such as a leathery appearance, dark/light pigmentation and deep furrows [2,3]. Natural aging is visible as wrinkling of the skin.

The skin is divided into three layers; the epidermis, dermis and subcutaneous tissue [4]. The extracellular matrix (ECM) is the outermost part of the skin and is composed of amongst others fibroblasts and proteins including collagen and elastin [5]. The ECM provides a structural framework which is essential for growth and elasticity of the skin and plays an important role in the maintenance of physiological functions of the body [5,6]. Degradation of the ECM has directly been linked to skin aging and is correlated with an increase in activity of certain enzymes involved in skin aging, which includes hyaluronidase, elastase and collagenase [3,7,8].

Collagen, one of the major building blocks of the skin, is the main component of connective tissue, hair and nails [1]. It is responsible for the elasticity and strength of the skin and maintains its flexibility. Hyaluronic acid plays a role in retaining the moisture of the skin, as well as its structure and elasticity. It also facilitates the exchange of nutrients and waste products and is involved in rapid tissue proliferation, regeneration and repair [9,10]. This compound is also involved in organisation and structural maintenance of the ECM [9]. With aging, collagen, elastin and hyaluronic acid levels decrease, leading to a loss of strength and flexibility in the skin which results in visible wrinkles.

Reactive oxygen species (ROS) play an important role in many cellular mechanisms [11]. When UV radiation is absorbed by the skin it leads to increased ROS generation and induction of oxidative stress. Oxidative damage may lead to lipid peroxide formation, mitochondrial and DNA damage, and protein and gene modification which alter protein structure and function [12]. High levels of ROS lead to the activation of hyaluronidase, collagenase and elastase, which can further contribute to skin aging [1,4,13].

Plants have long been used in the cosmetic industry as amongst others, skin lighteners and sun-screen agents. *In vitro* scientific studies have shown that plants possess the ability to reduce antioxidant levels and inhibit hyaluronidase, collagenase, elastase and tyrosinase activity [10,11,14].

People living on the African continent are exposed to the harsh sun and rarely use skin protective agents as they are expensive. It is for this reason that plants which are readily accessible be explored for their potential use as anti-aging reagents. Four plants were selected to assess their anti-collagenase, anti-hyaluronidase, anti-elastase and anti-oxidant activity. Traditionally, *Peltophorum africanum* (Fabaceae) is used to treat wounds [15], *Schotia brachypetala* to wash the body, reduce body swelling, steam the face, treat tropical ulcers and as an emetic for pimples [16], *Clerodendrum glabrum* to treat snakebites [17] and *Psychotria capensis* (Rubiaceae) to treat gastric complaints [18]. Selection of the plants was based on either its indigenous facial use; *S brachypetala,* its topical application to wounds; *P. africanum* or due to its good antioxidant activities exhibited in previous studies carried out in this laboratory (unpublished data); *C. glabrum* and *P. capensis*.

## Methods

### Chemicals and reagents

N-Methoxysuccinyl-Ala-Ala-Pro-Val-*p*-nitroanilide, human leukocyte elastase, 4-(2-hydroxyethyl)-1-piperazineethanesulfonic acid (HEPES, pH 7.5), sodium chloride, dimethysulfoxide (DMSO), N-Methoxysuccinyl-Ala-Ala-Pro-Chloro, elafin, N-[3-(2-furyl)acryloyl]-Leu-Gly-Pro-Ala (FALGPA), tris(hydroxymethyl)-methyl-2-aminoethane sulfonate (TES), calcium chloride dihydrate, ethylenediaminetetraacetic acid (EDTA), ninhydrin, citric acid, tin (II) chloride, 2-propanol, hyaluronic acid sodium salt from *Streptococcus equi*, bovine testicular hyaluronidase, potassium metaborate (KBO_2_), 4-dimethylaminobenzaldehyde (DMAB), sodium aurothiomalate, 2,2’-azinobis-3-ethyl benzothiazoline 6-sulfonic acid (ABTS), potassium peroxidisulfate and 6-hydroxy-2,5,7,8-tetramethylchroman-2-carboxylic acid (trolox) were procured from Sigma. Bovine serum albumin and collagenase type 1 from *Clostridium histolyticum* were purchased from Life Technologies, whereas methanol (MeOH) and ethyl acetate (EtOAc) were purchased from Merck.

### Plant collection and extract preparation

The plants investigated in this study are listed in Table 1. Specimens were collected by a botanist and the identity confirmed by the South African National Biodiversity Institute (SANBI, Tshwane) where voucher specimens are deposited. Voucher numbers are provided in Table 1.

**Table 1:** Plants, voucher number, plant parts and percentage yields of investigated specimens

**Plant species (Family)**	**Vernacular**	**Voucher number**	**Plant part**	**% Yield**	
				**MeOH**	**EtOAc**
*Clerodendrum glabrum* E. Mey var. *glabrum* (Verbenaceae)	Tinderwood	PRE 7554	Roots	4.99	1.77
			Stems	5.44	0.63
			Fruits	10.5	1.15
			Bark	5.19	1.00
*Peltophorum africanum* Sond. (Fabaceae)	African wattle	PRE 180	Leaves	14.7	1.37
			Seeds	9.56	3.58
			Bark	18.5	2.64
			Stems	7.49	1.03
*Psychotria capensis* (Eckl.) Vatke sub. *capensis* var. *capensis* (Rubiaceae)	Lemon bush	PRE 3047	Leaves	27.0	2.82
			Stems	7.00	0.46
			Seeds	11.9	1.60
*Schotia brachypetala* Sond. (Fabaceae)	African walnut	PRE 324	Bark	36.4	0.64
			Leaves	4.74	4.09

Plants were dried in an oven at 30-60°C and ground (1 mm) using a baby hippo hammer mill (A Collins & Son Pty. Ltd.). Ground plant material (20 g) was extracted with either EtOAc or MeOH using a Buchi Accelerated Speed Extractor E916. These solvents were selected based on reports of high extraction efficiency of antioxidants [19,20]. Resultant extracts were evaporated under vacuum at 40°C and stored at 4°C until use. The extract yields were determined gravimetrically (Table 1).

### Determination of anti-elastase activity

Anti-elastase activity was determined according to the method of Kraunsoe et al. [21], with minor modifications. Into 96-well plates was added: 25 μl each of 0.1 M HEPES buffer (pH 7.5), test sample (1.4 mg/ml) and elastase (1 μg/ml). The blank wells contained HEPES buffer (75 μl), the negative control 25 μl elastase and 50 μl HEPES buffer. The positive controls received 25 μl each of elastase, HEPES buffer and elafin/N-methoxysuccinyl-Ala-Ala-Pro-Chloro (10 μg/ml). The solvent controls contained 25 μl each of elastase, HEPES buffer and either 10% MeOH, 10% DMSO or 30% DMSO depending on the solvent the test sample was dissolved in. Extract controls containing 150 μl HEPES buffer and 25 μl of the extract were colour controls of each extract tested. Plates were incubated at room temperature for 20 min after which 100 μl of the substrate N-Methoxysuccinyl-Ala-Ala-Pro-Val-*p*-nitroanilide (1 mM) was added and the plates incubated for a further 40 min at 25°C. Absorbance was read at 405 nm using a Tecan Infinite 500 spectrophotometer. The percentage inhibition was calculated as follows:

$$\mathrm{Inhibition}\;\left(\%\right)=\left[\left(A_{\mathrm{control}}–A_{\mathrm{test}\;\mathrm{sample}}\right)/A_{\mathrm{control}}\right]\times 100$$

Where A_control_ is the absorbance of buffer, elastase + solvent and A_sample_ is the absorbance of buffer, elastase + extract or elafin/ N-Methoxysuccinyl-Ala-Ala-Pro-Chloro.

### Determination of anti-collagenase activity

The method of Moore and Stein [22] with modifications by Mandl et al. [23] was used to determine anti-collagenase activity. To 2 ml test tubes was added: 25 μl each of collagenase (1 mg/ml), TES buffer (50 mM) with 0.36 mM calcium chloride (pH 7.4) and test sample (1.4 mg/ml). The blank contained 75 μl TES buffer, while the negative control contained 25 μl collagenase and 50 μl TES buffer. The positive control contained 25 μl collagenase, 25 μl each of TES buffer and EDTA (1 mg/ml). The solvent control contained equal amounts (25 μl) of collagenase, TES buffer and either 10% MeOH, 10% DMSO or 30% DMSO depending on the solvent the test sample was dissolved in. The tubes were incubated in a water bath at 37°C for 20 min. Thereafter, 100 μl FALGPA was added to the tubes and incubated further for 60 min at 37°C. To all tubes, 200 μl of a solution containing equal volumes of 200 mM citrate buffer (pH 5) and ninhydrin solution was added. All tubes were placed in a water bath (100°C) for 5 min and left to cool to room temperature before adding 200 μL of 50% isopropanol to each tube. Contents in the tubes were then transferred to respective wells in 48-well plates. Absorbance was detected at 540 nm using a Tecan Infinite 500 spectrophotometer. The percentage inhibition was calculated using a formula similar to that in the previous section where A_control_ is the absorbance of buffer, collagenase + solvent and A_sample_ is the absorbance of buffer, collagenase + extract or EDTA.

### Determination of anti-hyaluronidase activity

The fluorimetric Morgan-Elson assay method of Reissig et al. [24] as modified by Takahashi et al. [25] was followed. Into 2 ml test tubes was placed: 25 μl of calcium chloride (12.5 mM), 12.5 μl each of test samples (2.8 mg/ml) and hyaluronidase (1.5 mg/ml). The blank contained 25 μl distilled water, the negative control 12.5 μl distilled water, the positive control 12.5 μl of sodium aurothiomalate (2.8 mg/ml) and the solvent control 12.5 μl of either 100% DMSO or MeOH. All the tubes except the blank received 12.5 μl of the enzyme. The tubes were incubated in a water bath (37^0^C; 20 min) after which 100 μl of the substrate hyaluronic acid (1 mg/ml in 0.1 M acetate buffer; pH 3.5) was added and the tubes incubated further for 40 min. Twenty-five microlitres KBO_2_ (0.8 M) was added to all tubes and the tubes were placed in a water bath (100°C) for 3 min, left to cool to room temperature and 800 μl of DMAB (4 g DMAB in 40 ml acetic acid and 5 ml 10 *N* HCl) was added. The tubes were then incubated for 20 min and the contents transferred to respective wells in a 48-well plate. Fluorescence was detected using a Tecan Infinite 500 spectrophotometer at 545 nm excitation and 612 nm emission. The percentage inhibition was calculated using the same formula provided earlier. Where A_control_ is the absorbance of buffer, hyaluronidase + solvent and A_sample_ is the absorbance of buffer, hyaluronidase + extract or sodium aurothiomalate.

### ABTS^•+^ radical scavenging activity

The trolox equivalent antioxidant assay was used to determine the ABTS^•+^ scavenging ability of the crude extracts, as reported by Re et al. [26]. Briefly, ABTS^•+^ (7.46 mM) was prepared in distilled water and oxidized using 2.5 mM potassium peroxidisulfate at 4°C for 16 h. The oxidized solution was diluted with distilled water to an absorbance of 0.70 ± 0.02 at 734 nm. Into a 96-well plate was added 20 μl Trolox (0; 0.0125; 0.04; 0.06; 0.075; 0.1 mg/ml) or 20 μl crude extracts (0.01; 0.032; 0.1; 0.32 and 1 mg/ml), and 180 μl ABTS^•+^. Plates were incubated for 15 min in the dark after which absorbance was measured at 405 nm using an ELx 800 UV universal microplate reader. The ABTS^•+^ scavenging capacity of extracts was compared to that of trolox and the percentage inhibition calculated using the following formula:

$$\begin{array}{l} \mathrm{ABTS}\;\mathrm{radical}\;\mathrm{scavenging}\;\mathrm{activity}\;\left(\%\right)\\ \;=\left[\left(A_{\mathrm{control}}–A_{\mathrm{sample}}\right)/A_{\mathrm{control}}\right]\times 100 \end{array}$$

A_control_ is the absorbance of ABTS^•+^ + solvent and A_sample_ is the absorbance of ABTS radical + extract or trolox.

### Statistical procedures

Tests were carried out in triplicate on two different occasions for the enzyme assays and on at least three occasions for the antioxidant assay. The results are presented as mean ± standard deviation (SD). IC_50_ values, which represent the concentration of the extract required to scavenge half the ABTS^•+^ radical, for the antioxidant assay were determined using GraphPad Prism Version 4.00 for windows.

The data for the enzyme assays was analyzed using MS Excel. The results are expressed as percentage inhibition. The percentage inhibitions were subjected to an appropriate analysis of variance (ANOVA) with the three enzymes as the main plot factor plus combinations of (the two solvents × six plant parts × four plant species × the positive controls) as sub-plot factors. The standardized residuals were tested for deviations from normality using the Shapiro-Wilk test [27]. The analyses were performed using SAS version 9.2 statistical software [28].

Research ethics forms were filled in and submitted to the ethics committee at the Council for Scientific and Industrial Research (South Africa). The committee approved the study stating that no ethics approval was required for this study.

## Results and discussion

*C. glabrum* (roots, stems, fruits and bark), *S. brachypetala* (leaves and bark), *P. africanum* (leaves, seeds, bark and stems) and *P. capensis* (leaves, stems and seeds) were investigated to determine the anti-aging potential of these plants with regards to anti-elastase, anti-hyaluronidase and anti-collagenase activity as well as antioxidant activity.

Statistical analyses of the results showed that deviations from normality (p < 0.001) were caused by Kurtosis not skewness, therefore the results were taken as normal [29]. From ANOVA the four factor interactions, mentioned in the statistical procedures section, were highly significant (p < 0.001). The mean percentage inhibitions are presented in Table 2 and the Student’s t.LSD (least significant difference) were calculated at 5% level of significance (t.LSD_p=0.05_ = 8.7146) to compare the means with a pooled variance of 16.5565 and 81 degrees of freedom.

**Table 2:** The effect of the plant extracts (200 μg/ml) on enzyme activities

**Plant species**	**Plant part**	**Extract type**	**Anti-elastase**	**Anti-collagenase**	**Anti-hyaluronidase**
*Clerodendrum glabrum*	roots	MeOH	5.92 ± 4.75	ng	ng
		EtOAc	15.30 ± 1.96	31.70 ± 0.54	ng
	stems	MeOH	13.35 ± 0.56	68.09 ± 8.57	ng
		EtOAc	89.38 ± 3.24	88.64 ± 0.53	8.11 ± 0.95
	fruits	MeOH	49.38 ± 3.82	72.54 ± 9.83	0.51 ± 0.72
		EtOAc	87.95 ± 0.10	26.29 ± 1.17	2.50 ± 3.53
	bark	MeOH	13.52 ± 6.96	58.96 ± 8.65	5.19 ± 1.61
		EtOAc	81.46 ± 4.78	81.55 ± 4.03	ng
*Peltophorum africanum*	leaves	MeOH	55.83 ± 0.04	82.13 ± 3.64	49.32 ± 6.58
		EtOAc	80.20 ± 1.02	75.26 ± 7.38	2.69 ± 3.80
	seeds	MeOH	85.17 ± 3.20	80.39 ± 9.18	ng
		EtOAc	87.09 ± 1.22	77.34 ± 4.45	2.18 ± 0.13
	bark	MeOH	69.36 ± 4.33	78.44 ± 3.18	48.46 ± 6.77
		EtOAc	86.27 ± 3.63	88.27 ± 1.77	23.24 ± 0.18
	stems	MeOH	64.50 ± 3.41	74.14 ± 1.08	0.95 ± 0.76
		EtOAc	84.40 ± 3.97	83.25 ± 3.57	13.78 ± 0.28
*Psychotria capensis*	roots	MeOH	45.90 ± 9.84	59.41 ± 6.73	12.86 ± 3.42
		EtOAc	59.60 ± 7.57	28.42 ± 5.23	2.28 ± 3.22
	stems	MeOH	42.98 ± 7.29	56.45 ± 9.26	5.00 ± 0.69
		EtOAc	56.58 ± 2.11	29.98 ± 0.81	ng
	leaves	MeOH	3.28 ± 4.63	ng	52.98 ± 0.66
		EtOAc	92.84 ± 1.13	63.07 ± 9.15	3.13 ± 4.42
	seeds	MeOH	44.16 ± 2.99	78.36 ± 1.50	ng
		EtOAc	70.67 ± 4.80	69.48 ± 5.91	ng
*Schotia brachypetala*	bark	MeOH	75.32 ± 6.91	77.51 ± 0.28	75.13 ± 7.49
		EtOAc	93.73 ± 0.51	87.61 ± 6.72	ng
	leaves	MeOH	82.54 ± 3.01	81.35 ± 2.52	20.36 ± 1.92
		EtOAc	84.22 ± 4.79	84.70 ± 1.33	6.29 ± 1.17
*Controls (10 μg/ml)*					
Elafin			93.09 ± 4.10	N/A	N/A
N-Methoxysuccinyl-Ala-Ala-Pro-Chloro			91.54 ± 4.14	N/A	N/A
EDTA			N/A	83.75 ± 2.89	N/A
Sodium aurothiomalate			N/A	N/A	100 ± 0.01

Results are given as percentage inhibition ± Standard Deviation, t.LSD (least significant difference)_p=0.05_ = 8.7146. Some extracts showed negligible enzyme activity (< 0.01%) and these are represented by ng, N/A represents not applicable.

### Anti-elastase assay

Elastin is a protein found in connective tissue which is responsible for the elasticity of the skin and lungs [5,6,30]. This protein is catalysed by the enzyme elastase. Degradation of elastin by intracellular elastase increases with age and/or repeated UV-radiation, leading to skin aging [5,6,13]. Twelve of the samples inhibited elastase by ≥ 80% (Table 2). The ethyl acetate extracts of *P. capensis* leaves (92.84 ± 1.13%) and *S. brachypetala* bark (93.73 ± 0.51%) had higher activity than N-Methoxysuccinyl-Ala-Ala-Pro-Chloro (91.54 ± 4.14%), and comparable activity to elafin (93.09 ± 4.10%). The solvents had a negligible effect on elastase activity.

To the knowledge of the authors, the elastase inhibitory activity of the four plants has not been reported before. However, the anti-elastase activity of plant species belonging to the same families as the studied plants has been reported. *Coffea arabica* (Fabaceae) leaf extracts have been reported to exhibit anti-elastase activity [28]. *Hedyotis diffusa* (synonym *Oldenlandia diffusa*, family Rubiaceae) has been found to inhibit human neutrophil elastase activity [31]. A phenanthrenedione, pterolinus K, and a chalcone (pterolinus L) from the heartwood of *Pterocarpus santalinus* (Fabaceae) were found to inhibit generation of the superoxide anion and the release of elastase [32] and iridoid glycoside esters isolated from the aerial methanol extract of *Ixora coccinea* (Rubiaceae) inhibited the release of elastase [33]. The anti-elastase activity of the family Verbanaceae has not been reported as yet.

### Anti-collagenase activity

Collagen, the major component of the skin, is degraded by the enzyme collagenase. Inhibition of collagenase activity delays the process of forming pre-collagen fibres and subsequently the wrinkling process [1]. Twenty-two extracts inhibited the enzyme by more than 50%, with nine of these inhibiting the enzyme by more than 80% (Table 2). The ethyl acetate extracts of *C. glabrum* stems and *P. africanum* bark and stems and *S. brachypetala* bark and leaves contained activity higher than the positive control, EDTA (83.75 ± 2.89%).

The anti-collagenase activity of the studied plants has not been reported previously. No information was found regarding the collagenase inhibition activity of plants belonging to the Verbanaceae and Rubiaceae families. In the Fabaceae family, *Coffea arabica* leaf extracts have been shown to inhibit collagenase-1 activity in a dose-dependent fashion [34].

### Anti-hyaluronidase activity

The methanol bark extract of *S. brachypetala* inhibited hyaluronidase activity the most, 75.13 ± 7.49%. Methanol leaf extracts of *P. capensis* and bark of *P. africanum* as well as ethyl acetate leaf extracts of this plant contained noticeable inhibitory activity (Table 2). The positive control, sodium aurothiomalate, completely inhibited the activity of hyaluronidase.

The anti-hyaluronidase activity of plants belonging to the Rubiaceae and Verbenaceae families has not been reported to date. The aqueous stem-bark extract of *Caesalpinia paraguariensis* (Fabaceae) has been reported to inhibit hyaluronidase [35]. Leaf extracts of *Astragalus membranaceus* are reported to increase the content of hyaluronic acid in cultured keratinocytes and fibroblasts by increasing mRNA expressions of hyaluronan synthase-3 and hyaluronan synthase-2 [10].

### Antioxidant activity

The concentration at which the extracts were able to scavenge half of the ABTS^•+^ radical (IC_50_) is presented in Table 3. The lower the IC_50_ value, the stronger antioxidant activity. Ethyl acetate extracts of the bark of *P. africanum* showed activity comparable to that of the positive control, trolox (IC_50_ 2.0 ± 0.23 μg/ml). Antioxidant activity has been reported for the acetone extract of the root and bark of *P. africanum*[15]. Other *Peltophorum* species with reported antioxidant activity include: *P. pterorcarpum, P. ferrugineum*, and *P. dubium*[36-38].

**Table 3:** Antioxidant activity of tested extracts

**Plant name**	**Plant part**	**Extract type**	**IC**_**50**_/(μg/mL)*
*Clerodendrum glabrum*	Roots	MeOH	21.1 ± 1.72
		EtOAc	17.8 ± 6.47
	Stems	MeOH	17.0 ± 1.96
		EtOAc	9.90 ± 3.27
	Bark	MeOH	10.8 ± 0.99
		EtOAc	ng
	Fruits	MeOH	35.5 ± 4.01
		EtOAc	63.9 ± 5.03
*Peltophorum africanum*	Leaves	MeOH	5.3 ± 0.72
		EtOAc	9.2 ± 0.76
	Seeds	MeOH	7.16 ± 0.93
		EtOAc	11.4 ± 2.48
	Bark	MeOH	4.9 ± 0.47
		EtOAc	2.0 ± 0.23
	Stems	MeOH	4.5 ± 0.38
		EtOAc	2.1 ± 0.17
*Psychotria capensis*	Stems	MeOH	11.3 ± 1.90
		EtOAc	52.0 ± 15.30
	Roots	MeOH	9.6 ± 0.73
		EtOAc	9.5 ± 1.06
	Leaves	MeOH	68.3 ± 8.87
		EtOAc	62.3 ± 6.00
	Seeds	MeOH	25.8 ± 2.72
		EtOAc	23.9 ± 4.57
*Schotia brachypetala*	Bark	MeOH	2.8 ± 0.31
		EtOAc	7.7 ± 0.55
	Leaves	MeOH	7.8 ± 0.63
		EtOAc	50.3 ± 9.33
Trolox	N/A	N/A	2.84 ± 0.52

*Results are given as IC_50_ ± SD ng = extracts had negligible antioxidant activity.

Extracts of *C. glabrum* contained moderate antioxidant activity. Antioxidant activity and free radical scavenging activity has been reported for various *Clerodendrum* species: *C. infortunatum*[39], *C. siphonathus*[40] and *C. glandulosum*[41]. The antioxidant activity noted for the methanol and ethyl acetate bark extracts of *S. brachypetala* is supported by studies where antioxidant and hydroxyl radical scavenging activity have been reported for the aqueous bark extracts of this plant [42,43]. Isolated compounds from this plant with known antioxidant activity include stilbenes and phenolics [44].

The root extracts of *P. capensis* contained antioxidant activity < 10 μg/ml. Although the antioxidant activity of this plant species has not previously been reported, the antioxidant activity of extracts from other *Psychotria* species are known; *P. brachyceras, P. umbellata*, *P. serpens*, and *P. rostrata*[45-47].

## Conclusion

This is the first study to investigate the anti-elastase, anti-collagenase and anti-hyaluronidase activity of *C. glabrum*, *P. capensis*, *P. africanum* and *S. brachypetala*. The free radical scavenging activity and enzyme inhibitory activity of the plant extracts suggests that they can help restore skin elasticity and thereby slow the wrinkling process. *P. africanum* was the plant with the most promising activity and will be subjected to further testing and isolation of the active compound/s.

## Abbreviations

DMSO: Dimethysulfoxidel; FALGPA: N-[3-(2-Furyl)acryloyl]-Leu-Gly-Pro-Ala; TES: Tris(hydroxymethyl)-methyl-2-aminoethane sulfonate; EDTA: Ethylenediaminetetraacetic acid; KBO2: Potassium metaborate; DMAB: 4-dimethylaminobenzaldehyde; ABTS: 2,2’-azinobis-3-ethyl benzothiazoline 6-sulfonic acid; trolox: 6-hydroxy-2,5,7,8-tetramethylchroman-2-carboxylic acid; MeOH: Methanol; EtOAc: Ethyl acetate.

## Competing interests

The authors declare that they have no competing interests.

## Authors’ contributions

GN was involved in both the antioxidant and enzyme assays and in drafting the manuscript. WC was involved in the ABTS including preparing reagents, running and analysing the data. MT helped with running and analysis of the enzyme assays. VS and GF supervised this study and revised the manuscript. All authors have read, corrected and approved the final manuscript.

## Pre-publication history

The pre-publication history for this paper can be accessed here:

http://www.biomedcentral.com/1472-6882/13/304/prepub
